# Supplementary material for: Gender Differences among Nurses in Managing Arterial Puncture-Related Pain: A Multicenter Cross-Sectional Study
Source: Healthcare (Basel). 2024 Feb 23;12(5):531. doi: 10.3390/healthcare12050531 (PMC10931307; doi:10.3390/healthcare12050531)

Supplementary Materials

Figure S1. Comparison in the perception of pain produced by ABG puncture according to pain reduction interventions and gender.

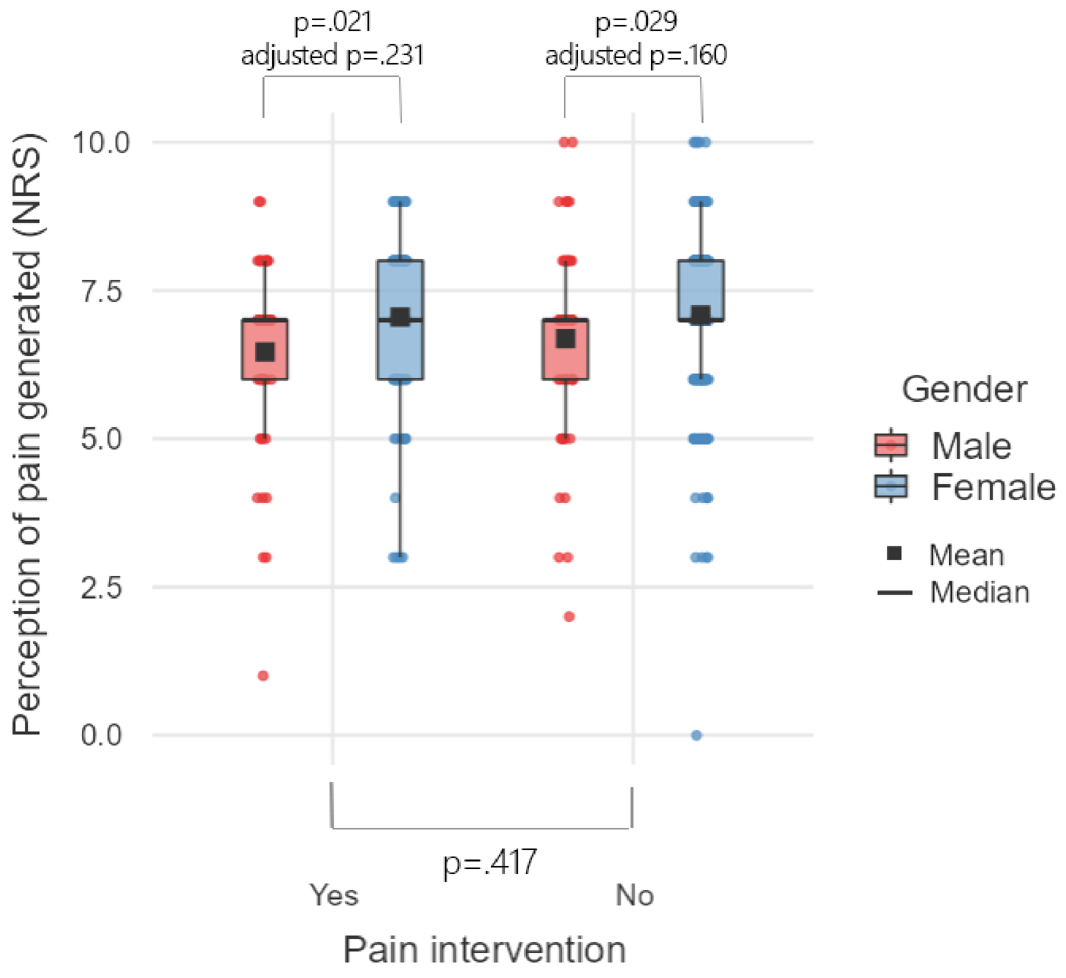

Figure S2. Discriminative ability represented by the AUCROC curve of the multivariable explanatory model of the use of pain-reducing interventions where gender is adjusted for other factors.

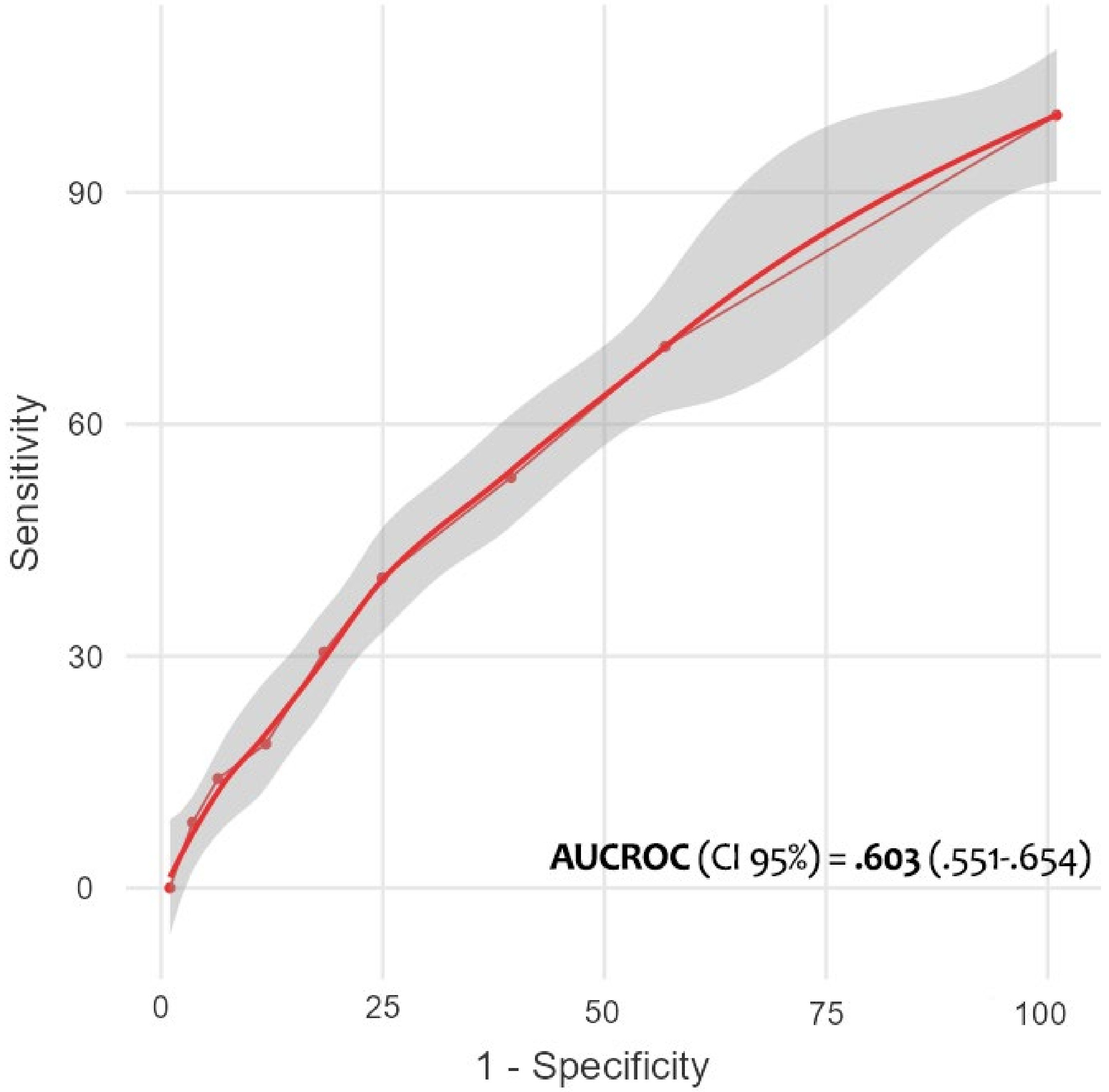

Supplement: Supplementary file 1 [file healthcare-12-00531-s001.zip › healthcare-2818644-supplementary.pdf]
